# The British Orthopaedic Surgery Surveillance study: slipped capital femoral epiphysis: the epidemiology and two-year outcomes from a prospective cohort in Great Britain

**DOI:** 10.1302/0301-620X.104B4.BJJ-2021-1709.R1

**Published:** 2022-04-01

**Authors:** Daniel C. Perry, Barbara Arch, Duncan Appelbe, Priya Francis, Joanna Craven, Fergal P. Monsell, Paula Williamson, Marian Knight, on behalf of the BOSS collaborators

**Affiliations:** 1 Faculty of Health and Life Sciences, University of Liverpool, Liverpool, UK; 2 Trauma and Orthopaedics Department, Alder Hey Children's Hospital, Liverpool, UK; 3 Kadoorie Centre, Oxford Trauma and Emergency Care, NDORMS, University of Oxford, Oxford, UK; 4 North West School of Surgery, Health Education England, Liverpool, UK; 5 Bristol Royal Hospital for Children, Bristol, Bristol, UK; 6 National Perinatal Epidemiology Unit, University of Oxford, Oxford, UK

**Keywords:** Cohort study, Epidemiology, Incidence, BOSS, SCFE, Slipped capital femoral epiphysis, Slipped upper femoral epiphysis, Avascular necrosis, SUFE, Slipped capital femoral epiphysis (SCFE), orthopaedic surgery, hip(s), avascular necrosis, Patient-reported outcome measures (PROMs), clinicians, randomized controlled trials, deformity, prophylactic fixation, open reduction and internal fixation

## Abstract

**Aims:**

The aim of this study was to inform the epidemiology and treatment of slipped capital femoral epiphysis (SCFE).

**Methods:**

This was an anonymized comprehensive cohort study, with a nested consented cohort, following the the Idea, Development, Exploration, Assessment, Long-term study (IDEAL) framework. A total of 143 of 144 hospitals treating SCFE in Great Britain participated over an 18-month period. Patients were cross-checked against national administrative data and potential missing patients were identified. Clinician-reported outcomes were collected until two years. Patient-reported outcome measures (PROMs) were collected for a subset of participants.

**Results:**

A total of 486 children (513 hips) were newly affected, with a median of two patients (interquartile range 0 to 4) per hospital. The annual incidence was 3.34 (95% confidence interval (CI) 3.01 to 3.67) per 100,000 six- to 18-year-olds. Time to diagnosis in stable disease was increased in severe deformity. There was considerable variation in surgical strategy among those unable to walk at diagnosis (66 urgent surgery vs 43 surgery after interval delay), those with severe radiological deformity (34 fixation with deformity correction vs 36 without correction) and those with unaffected opposite hips (120 prophylactic fixation vs 286 no fixation). Independent risk factors for avascular necrosis (AVN) were the inability of the child to walk at presentation to hospital (adjusted odds ratio (aOR) 4.4 (95% CI 1.7 to 11.4)) and surgical technique of open reduction and internal fixation (aOR 7.5 (95% CI 2.4 to 23.2)). Overall, 33 unaffected untreated opposite hips (11.5%) were treated for SCFE by two-year follow-up. Age was the only independent risk factor for contralateral SCFE, with age under 12.5 years the optimal cut-off to define ‘at risk’. Of hips treated with prophylactic fixation, none had SCFE, though complications included femoral fracture, AVN, and revision surgery. PROMs demonstrated the marked impact on quality of life on the child because of SCFE.

**Conclusion:**

The experience of individual hospitals is limited and mechanisms to consolidate learning may enhance care. Diagnostic delays were common and radiological severity worsened with increasing time to diagnosis. There was unexplained variation in treatment, some of which exposes children to significant risks that should be evaluated through randomized controlled trials.

Cite this article: *Bone Joint J* 2022;104-B(4):519–528.

## Introduction

Slipped capital femoral epiphysis (SCFE) is a disease of the adolescent hip joint that affects one in 1,300 adolescents.^1,2^ The mechanism is principally failure of the physis in response to load. Childhood obesity appears to be the primary cause of SCFE.^1,2^ In the short term, it always requires urgent surgery to stabilize the physis, and typically results in deformity of the hip. In the short term, this leads to damage to the acetabular cartilage.^3^ In the long term, SCFE accelerates the development of osteoarthritis, and many patients have disability necessitating hip arthroplasty in early adulthood.^4^ SCFE typically has an insidious onset, presenting to the family doctor, emergency department, or physiotherapist with knee, thigh, or hip pain, and/or a limp.^1^ In many patients the diagnosis is overlooked, and it is common for the diagnosis to take several months.^1,5^

While SCFE is the most frequent hip disease of adolescence, robust evidence to support effective management and intervention is poor, with no clinical trials to guide treatment decisions. Information regarding outcomes is almost universally limited to retrospective case series arising from single centres. Poor-quality evidence has contributed to considerable differences in surgical practice internationally.^6-8^ This has prompted members of the British Society of Children’s Orthopaedic Surgery to prioritize questions pertaining to SCFE among the top five research priorities in children’s orthopaedic surgery.^9,10^

Criticism within *The Lancet* of the poor quality of evidence in surgery^11^ prompted the development of a framework to guide surgical research, called the Idea, Development, Exploration, Assessment, Long-term study (IDEAL) framework.^12-14^ We describe a national cohort study of SCFE that follows the IDEAL recommendations (IDEAL Stage 2b, ‘Exploration’), to explore the disease frequency, case mix, technical intervention variables, surgeon- and patient-reported outcomes, and safety.

## Methods

We prospectively identified a cohort of skeletally immature children with a new radiological diagnosis of SCFE undergoing surgery in Great Britain. Children were identified between 4 April 2016 and 30 September 2017. Data were collected through a national surveillance programme, called the British Orthopaedic Surgery Surveillance (BOSS) study. This used a bespoke electronic platform. A full protocol detailing the BOSS study platform and details of the SCFE study has been previously published.^15,16^ All but one hospital in Britain that routinely treated SCFE agreed to participate in the study (n = 143). The denominator population was defined as six- to 18-year-olds, within the geographical boundaries of Great Britain.

Children were identified prospectively by treating clinical teams. Following admission to hospital, nominated clinicians completed an electronic patient record form providing anonymous details related to the patient, disease presentation, and surgery. Patients who were potentially missed were identified monthly by cross-referencing collected data with routinely collected administrative data available from Hospital Episode Statistics (HES) for England, Patient Episode Database for Wales (PEDW), and the Information Services Division (ISD) for Scotland. Missing patient records were also available from an independent network of trainee surgeons. Each month an automated email was sent to nominated reporting clinicians in each hospital to ask them to verify and confirm the completeness of their monthly case uploads (including verification of a null report). In addition, the email highlighted potential missed patients identified through administrative data or by trainees, and invited reporting clinicians to upload the missing records or indicate these as a false identification. The email also identified when follow-up was due. Surgeon follow-up from routine care records was sought at three months and two years post-surgery to identify complications, principally the development of avascular necrosis (AVN), chondrolysis, reoperations, and contralateral disease.

Patient-reported outcome measures (PROMs) were collected for a subset of hospitals recruiting patients to the study. While surgeons entered patient data to collect anonymized outcomes from the time of diagnosis, patients could be enrolled to provide PROMs at any time during the two years of follow-up. Regardless of when patients were enrolled, PROMs were collected following the schedule related to the date of surgery. The PROMs collected were the Paediatric Quality of Life Inventory (PedsQL),^17^ the youth version of the EuroQol five-dimension three-level questionnaire (EQ-5D-Y),^18^ and the Wong-Baker FACES Pain Rating Scale.^19^

The study was registered on the ISCTRN registry (ISRCTN54477575). The study is reported in accordance with the IDEAL reporting guidelines.^20^ Parents and representatives from STEPS Worldwide, the primary charitable support group for children and young people with limb disorders, coproduced the work from prefunding until publication with involvement in producing patient-facing materials and defining outcomes and outcome timepoints. A parent representative participated in the Study Management Group throughout the five-year study period. The Alder Hey NIHR Young Person’s Advisory Group (YPAG), a group of children and young people involved in improving the conduct of research in children, were also involved throughout the progress of the study. The YPAG have assisted in the development of the paper and digital participant information materials, advised on conduct during interval study updates, and are engaged in the dissemination of this work to patients and the public through animations and infographics.

### Statistical analysis

The statistical analysis was finalized prior to data lock. Incidence rates for new SCFE (no prior diagnosis in either hip) were calculated using patients identified between 1 June 2016 and 31 August 2017, which allowed for a ‘run-in period’ in terms of case ascertainment and discounted the final month of data collection for which national administrative data were unavailable. Rates were calculated by country, region, age, and sex. Denominators were taken to be the 2016 mid-year Office for National Statistics population estimates.^21^ Poisson 95% confidence intervals (CIs) were calculated.

Cohort characteristics were summarized, as well as medical history, timeline prior to surgery, imaging availability, and presentation of affected hip(s). Patients were classified according to the commonly used definition of clinical stability (i.e. ‘stable’ is when a child is able to walk at the time of admission, with or without the use of crutches),^22^ and hips were classified by the radiological severity of SCFE determined by the treating surgeon according to their usual practice (i.e. mild, moderate, or severe). Further detail was summarized regarding presentation, the use of prophylactic surgery in the unaffected (opposite) hip, fixation techniques, and postoperative planning. Full details of pre-planned analysis related to cohort characteristics is available in Supplementary Material.

Over the course of a two-year follow-up, we summarized the complications for affected hips, and unaffected (opposite) hips. Random effects logistic regression was used to model the risk of AVN in all affected hips, adjusting for stability of hip at presentation and use of open reduction, and in open reduction, adjusting for surgeon experience. The results of multivariable modelling, in line with the statistical analysis plan, can be found in the full statistical analysis report (Supplementary Material).

The effects of patient and treatment factors on the risk of contralateral SCFE, in unilateral first presentations without prophylactic surgery, were explored using survival analysis (Kaplan-Meier graphs and Cox proportional hazards modelling). Recursive partitioning was also used to generate an understanding of key binary predictors of this outcome. A separate analysis was carried out investigating PROMs. Given the low numbers of patients for this analysis, results are presented using descriptive statistics. We have chosen to calculate percentages throughout, excluding missing data. Tables in the full BOSS statistical analysis report indicate numbers missing for each variable (Supplementary Material).

## Results

Overall, 486 children had a new radiological diagnosis of SCFE identified during the 18-month recruitment period. Patients were identified from 100 of the 143 recruiting hospitals, with the median number of patients in each hospital being two (interquartile range (IQR) 0 to 4). Of these, 17 children were excluded from follow-up analyses as baseline case report forms were not completed beyond initial case confirmation, leaving a cohort of 469 children (513 hips).

### Disease frequency

The annual incidence of SCFE was 3.34 (95% CI 3.01 to 3.67) patients per 100,000 six- to 18-year-olds throughout the incident period. Rates were broadly similar across England, Scotland, and Wales (Table I).

**Table I.** Annual incidence of slipped capital femoral epiphysis stratified by country, region, age, and sex between 1 June 2016 and 31 August 2017.

Variable	Population, n	First presentation	
		n	Incidence (95% CI)
All	9,499,724	397	3.34 (3.01 to 3.67)
**Region**			
**England**	8,301,394	357	3.44 (3.08 to 3.80)
London/surrounding boroughs	1,851,204	81	3.5 (2.78 to 4.35)
South	1,696,467	69	3.25 (2.53 to 4.12)
Northern	2,280,272	99	3.47 (2.79 to 4.16)
Central	2,473,451	108	3.49 (2.83 to 4.15)
**Wales**	454,551	7	1.23 (0.50 to 2.54)
**Scotland**	743,779	33	3.55 (2.44 to 4.98)
**Age group**			
6 to 10 yrs	3,850,071	79	1.64 (1.30 to 2.05)
11 to 18 yrs	5,649,653	318	4.5 (4.01 to 5.00)
**Sex**			
Male	4,867,679	225	3.7 (3.21 to 4.18)
Female	4,632,045	172	2.97 (2.53 to 3.41)

* Six- to 18-year-olds in England, Scotland, and Wales (2016 mid-year estimate from the Office for National Statistics).

CI, confidence interval.

### Sensitivity analysis using administrative data

International Classification of Diseases-10^23^ and OPCS Classification of Interventions and Procedure codes^24^ within routine hospital administrative data from England, Scotland and Wales identified 596 admission records of SCFE within the incident period. Welsh data was not provided after 1 April 2017. Of these 596 records, clinicians confirmed that 222 of these records were not new disease episodes (i.e. previously diagnosed disease undergoing secondary procedures) and six records were duplicates. In total, 336 (91.3%) of the remaining 368 codes matched to new patients within the database using the predefined parameters (i.e. month and year of birth, sex, date of surgery, and hospital), leaving 32 unmatched. There were an additional 61 patients confirmed by clinicians, for which there was no record within administrative data. Within the administrative data, the 32 unmatched codes were unresolved at the time that the database closed for case ascertainment. Assuming that the 32 patients who were all missed were true, the incidence would rise to 3.61 (95% CI 3.27 to 3.95) cases per 100,000 six- to 18-year-olds.

### Participant characteristics

The median age of children was 12.7 years (IQR 11.4 to 13.8), with a slight preponderance in males (Table I). Of the 486 participants, 149 were recorded as having at least one comorbidity. The principal comorbidity being obesity (123 children; 26.3%), with others including hypothyroidism (nine children; 1.9%), other endocrinopathies (seven children; 1.5%), Down’s syndrome (four children; 0.9%), and prior treatment with radiotherapy (one child; 0.2%).

### Disease characteristics

A total of 102 children (21%) were described to have a clinically unstable SCFE, with the child unable to walk with or without the use of crutches at diagnosis. Among the 380 children (402 hips) with stable SCFE, the deformity was described as mild in 215 hips (53%), moderate in 109 (27%), and severe in 75 (19%).

### Diagnostic journey

In 344 of the 486 individuals, clinicians indicated more than one week elapsed from the onset of symptoms to admission for treatment. There were 311 free-text entries describing events during this time interval: 98 highlighted delays by the family in seeking healthcare; 96 identified multiple healthcare attendances with an initial diagnosis of “groin sprain”, “growing pains”, or similar; 28 reported radiographs were initially believed to be normal; and 26 noted that the radiologically confirmed disease was referred on a non-urgent basis. Other delays included delays accessing radiographs or delays in accessing specialist opinions prior to a definitive diagnosis. Among stable slips, the disease severity increased as the median time to diagnosis increased (Table II).

**Table II.** Clinical timeline illustrating the patient journey from onset of symptoms to diagnosis, stratified by slipped capital femoral epiphysis stability.

Variable	Stable at presentation			Unstable at presentation
	Mild	Moderate	Severe	
Hips, n	176	90	69	94
Median time to diagnosis, mths (IQR)	1 (0 to 2)	2 (1 to 8)	3 (0 to 7)	1 (0 to 2)

IQR, interquartile range.

### Surgical strategy

**Fig. 1:**
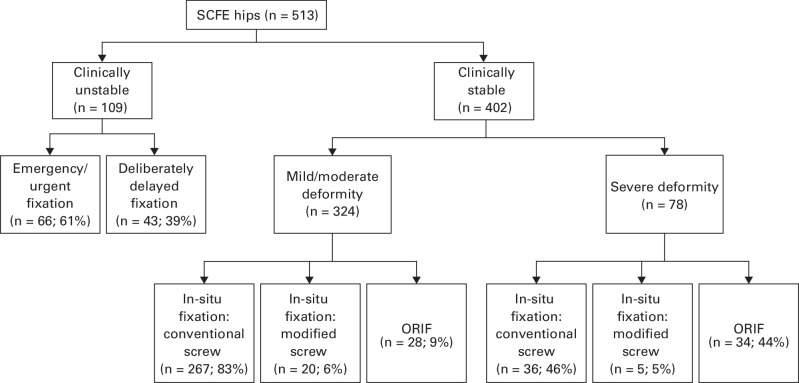
Surgical management according to the clinical stability and radiological severity at baseline. ORIF, open reduction and internal fixation; SCFE, slipped capital femoral epiphysis.

Surgical fixation involved the use of a single screw to stabilize the physis in 433 hips (85%), with multiples screws used in 61 hips (12%). Ten hips (2%) were stabilized without screws, while 25 hips were treated with modified screws designed to permit growth at the physis. Among patients for whom the slip was classified stable, surgery was generally undertaken as planned surgery on the next available routine trauma list (299/380; 79.9%). Among patients for whom the slip was classified unstable, a planned delay to surgery occurred in 37/96 (38.5%), at a median of 8.5 days (IQR 5.5 to 10.0). Open reduction to reduce the severity of the SCFE was undertaken in 95 hips (53 unstable, 41 stable, 1 unreported), of which 39 were performed through a surgical dislocation and 51 performed without dislocating the hips. The technique was unreported in five hips. The clinical stability and radiological severity of the hip strongly influenced the surgical strategy (Figure 1).

There were 406 unaffected hips (i.e. patients for whom the opposite hip was not, or had not previously been affected, by SCFE). Of these, 120 (30%) underwent prophylactic fixation to prevent SCFE, leaving 286 (70%) at risk of SCFE. Of those undergoing prophylactic fixation, surgeons identified this as their standard protocol in 57 hips (48%), while others cited risk factors of obesity, younger age, or ‘other’ as the reason for fixation.

### Outcomes

Outcomes were recorded for 89% of hips at three months and 79% at two years. Data were missing either because patients were identified as lost to follow-up (20 hips at three months and 62 hips at two years), or because patient record forms were not returned by clinicians (34 hips at three months, 47 hips at two years).

### AVN

Overall, 29 hips (7.1%) were affected by AVN by two years. Nine patients with AVN occurred within the group of 316 clinically stable hips with SCFE. Among stable hips for which open reduction was performed, 4/34 (11.8%) developed AVN, whereas AVN occurred in 5/276 (1.8%) stable hips that did not undergo open reduction. AVN occurred in 20/90 hips (23.0%) presenting with ‘unstable’ SCFE. The ORs of AVN were 4.4 (95% CI 1.7 to 11.4) for unstable versus stable hips, and 7.5 (95% CI 2.4 to 23.2) for hips undergoing open reduction versus not (Table III). Surgeon-reported experience in the technique of open reduction did not appear to mitigate the risk of AVN (Table IV).

**Table III:** Risk factors for the development of avascular necrosis, Model 1: risk of avascular necrosis adjusted for baseline stability and open reduction of the hip (397 hips from 363 children). All models include a random effect for child to allow bilateral presentations to be included.

Covariate	Hips, n	Any AVN, n (%)	OR (95% CI)
**Stability of hip at presentation**			
Stable	310	9 (2.9)	1.0 (reference)
Unstable	87	20 (23.0)	4.4 (1.7 to 11.4)
**Open reduction carried out at baseline**			
Yes	79	21 (26.7)	7.5 (2.4 to 23.2)
No	318	8 (2.5)	1.0 (reference)
**Hips excluded from model**	116		
Stability/open reduction status missing	11		
Followed up, but AVN status unknown	85		
Lost to follow-up	20		

AVN, avascular necrosis; CI, confidence interval; OR, odds ratio.

**Table IV.** Risk factors for the development of avascular necrosis, Model 2: risk of avascular necrosis in open reduction adjusted for experience of most senior surgeon (72 hips with open reduction from 68 children). All models include a random effect for child to allow bilateral presentations to be included.

Covariate	Hips, n	Any AVN, n (%)	OR (95% CI)
Experience of most senior surgeon*	72	19 (26.4)	1.0 (0.8 to 1.2)
0	5	2 (40)	
1	7	2 (29)	
2	12	4 (33)	
3	10	3 (30)	
4	6	0 (0)	
5 to 10	13	2 (15)	
> 10	19	6 (32)	
**Hips excluded from model**	23		
Experience of surgeon missing	12		
Followed up, but AVN status unknown	7		
Lost to follow-up	4		

* Number of similar procedures using the open reduction technique performed in past year by the senior surgeon.

AVN, avascular necrosis; CI, confidence interval; OR, odds ratio.

### Other complications

Chondrolysis occurred in three hips (0.8%) by two years. Other complications included surgical site infection (n = 4; 0.9%), osteomyelitis (n = 1; 0.2%), periprosthetic fracture (n = 2; 0.4%), implant penetration into the joint (n = 6; 1.3%), and screw failure or movement resulting in disease recurrence (n = 5; 1.1%). Following the initial surgery, 74 hips (16%) were known to have undergone secondary surgery by two years, with a further 43 (11%) scheduled to have surgery in the period after completion of the study. Most notably, 11 (2.5%) had undergone (or were scheduled to undergo) total hip arthroplasty, and ten (2.2%) had undergone, or were scheduled to undergo, major realignment surgery involving corrective osteotomies.

A preplanned analysis using α angle to describe hip shape was undertaken, though yielded little usable information. These are available within the Supplementary Material.

### Opposite (unaffected) hips

Among the 120 hips that underwent prophylactic fixation, follow-up was available in 117 (97.5%) at three months and 106 (88.3%) at two years. By three months, four (3.6%) developed a surgical site infection, one (0.9%) was revised for a periprosthetic fracture, and one (0.9%) for implant penetration into the joint. By two years, one (1.0%) developed AVN, and one (1.0%) developed osteomyelitis, with additional surgery undertaken on a further eight hips, composed of two (1.7%) exchange/adjustment of screws, one (0.9%) failed removal of screw, and five (4.3%) removal of screws. No hips that had been prophylactically fixed developed a subsequent SCFE.

**Fig. 2:**
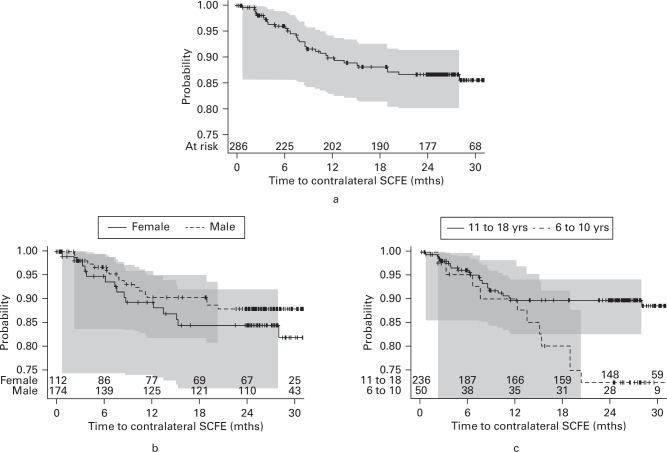
Kaplan-Meier plots with 95% confidence intervals illustrating the probability of remaining disease-free in a) the opposite hip, stratified by b) sex and c) age. SCFE, slipped capital femoral epiphysis.

In total, 286 hips unaffected by SCFE did not undergo prophylactic fixation. Among these, 33 operations for SCFE were recorded during the two-year follow-up period (11.5% (95% CI 7.8% to 15.2%)). The Kaplan-Meier plot illustrates the time from the start of the at-risk period (the time of first SCFE surgery) to diagnosis, stratified by sex and age (Figures 2a to c). A univariate analysis demonstrated that age appeared to be an important determinant of disease, with younger children at greater risk of SCFE. Other baseline predictors (sex, BMI, SCFE severity) were not predictors of subsequent contralateral disease.

A decision tree analysis, using recursive partitioning, showed that age alone was the most useful predictor of contralateral disease: the optimal partition was at 12.5 years, with 23 patients from 124 hips (18.5%) below this age, and nine patients from 164 hips (5.6%) above this age.

### PROMs

A nested cohort of participants (n = 144) consented to complete PROMs and could be recruited at any point during the follow-up period. In total, 50 of the 143 sites agreed to collect PROMs: 47 participants completed PROMS at baseline, from 49 participants enrolled at this timepoint. However, only 59 patients completed outcomes at two years (41% of those enrolled). The baseline demographic data of those completing PROMs were broadly similar in terms of age, sex, ethnicity, BMI, and clinical stability to those in the surveillance cohort (Supplementary Material). However, a slightly larger proportion of those completing PROMs were classified as having more severe SCFE, with 26/59 (44%) having severe deformity and 14/59 ( 24%) moderate deformity.

**Fig. 3:**
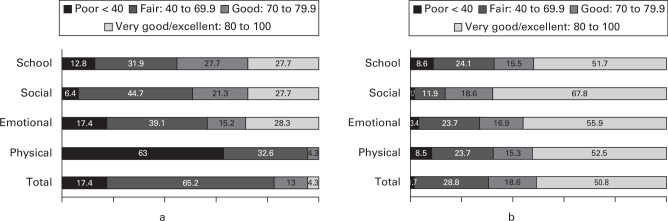
Distribution of Paediatric Quality of Life Inventory in slipped capital femoral epiphysis patients at a) baseline (n = 47), and b) two years (n = 59). Categorizations defined by Rosenberg et al.^25^

At diagnosis, SCFE had a marked impact on health, which was particularly evident within the domain of physical health (PedsQL median total score 52.7 (IQR 43.5 to 66.3) and median physical health score 31.3 (IQR 15.6 to 50.0)). At two years, the median total score was 82.6 (IQR 63.0 to 92.4) and median physical health score was 83.3 (IQR 62.5 to 90.6). Overall, 51% participants (30/59) reported ‘very good or excellent’ health by two years, compared to 4.3% (2/47) at baseline (Figure 3).^25^ This was reflected in the EQ-5D-Y scores, which were 0.1 (IQR -0.2 to 0.6) at baseline, improving to 0.9 (IQR 0.8 to 0.1) at final follow-up. Similarly, there were improvements in pain. However, questionnaire completion was insufficient to investigate the association between PROMs and baseline factors.

## Discussion

This is the most comprehensive study to investigate SCFE, with almost complete involvement of hospitals throughout Britain. We identified the rarity of SCFE in hospitals, with the median number of patients encountered over the study period per hospital of two. We found an association between time to diagnosis and the severity of disease, coupled with recognizing the barriers to making a prompt diagnosis. We also demonstrated marked differences in surgical decisions regarding the treatment of similar patients with SCFE, with evidence that this has important effects on patient outcomes and the complications that affected children encounter.

The comprehensive case-finding mechanism enabled a reliable estimate of incidence, with few patients being identified within routine nationwide hospital administrative data unaccounted for. The slightly lower rate of disease in Wales may be accounted for with incomplete access to administrative data in the later period.

The study particularly highlights the variation in practice. Uncertainty was notably related to three areas of surgical practice: clinically unstable hips, with 60% having urgent surgery, while 40% had surgery that was deliberately delayed by at least one week; clinically stable hips with severe deformity, for which half of the children had correction of the deformity and half had fixation of the deformity without correction; and unaffected opposite hips, of which 30% were operated on to prevent SCFE, and 70% were not. This variation is in part due to a lack of evidence from randomized controlled trials (RCTs) to support any element of practice in SCFE surgery, with interventions often having major variation in the magnitude and complexity of surgery.

This cohort confirms the high risk of AVN among unstable hips, the magnitude of which was broadly in keeping with other studies.^26^ Among unstable hips, a particular difference in practice was that some surgeons enforced a period of ‘bed rest’ prior to surgery, in the belief that there is a time window (between 24 hours and one to two weeks) during which surgery heightens the risk of AVN.^27,28^Among those patients with severe deformity, surgeons are generally divided on whether to perform open reduction to normalize the anatomy, albeit risking AVN, or to accept deformity and the symptoms and potential sequelae associated with it. A review by the UK National Institute for Health and Care Excellence noted the safety risk related to AVN, and concluded that the procedure should only be undertaken with special arrangements for governance, consent, audit, and research.^29^ We have demonstrated that open reduction carries a high risk of AVN in SCFE, independent of stability at baseline, with no evidence that this risk is mitigated by the experience of the surgeon. However, the benefit of surgery on the physical function of the child may be sufficient to outweigh the risks, the answer to which is not available from this study.

For the unaffected (opposite) hip, we have also shown that the importance of age in the risk of SCFE, which appears likely to be related to the ‘at risk period’ (i.e. years of growth) remaining. Prophylactic surgery did prevent the development of SCFE but also had several major complications, including one patient who developed AVN and a subtrochanteric fracture, which must be balanced against the risk of SCFE in this group. A sensible approach appears to be one that considers individual risk; however, the only baseline predictor contributing to future risk was age, with younger children having greater risk, especially younger than 12.5 years old. Other factors, such as sex, BMI, clinical stability, or radiological severity, did not contribute to the risk. We are aware that there may be other factors to consider that we did not record, such as biochemical measures (i.e. leptin) or specific radiological measures (i.e. epiphyseal tubercle anatomy).^30,31^

The collection of PROMs demonstrated the profound impact of SCFE on affected children in the immediate aftermath of disease, with the impact on life still persisting two years after the diagnosis. The biggest impact to the children was on their physical health, although all health domains were impacted by disease.

As well as highlighting areas of controversy within surgical practice, this study highlighted areas with little controversy, such as the need to use a single conventional screw for the majority of patients. In addition, we identified obesity as the major comorbidity among those with SCFE. Other comorbid diseases that are commonly believed to be risk factors (i.e. hyopthyroidism) were rare in this population.^1,2^

Challenges in receiving a prompt diagnosis were evident. There was poor awareness of the disease among healthcare professionals and families. Furthermore, even when a diagnosis was made, treatment was not always recognized to be urgent, with missed diagnostic opportunities evident among physiotherapists, radiologists, family doctors, emergency doctors, and orthopaedic surgeons. Delay was important, because we identified that an increasing delay was associated with a worsening radiological severity of disease at diagnosis. While education will inevitably be the mainstay of raising awareness of SCFE and its treatments, the use of technology may offer opportunities. Diagnostic prompts may be used in electronic health records to flag ‘at risk’ children with relevant symptoms, and machine vision may be used to flag radiographs that the computer identifies to show SCFE.

A strength of this study was the completeness of case ascertainment, which minimized the likelihood of selection bias. The incidence of disease appears likely to be reliable, with only one small hospital not contributing to the study, where there were no patients with SCFE identifed from their hospital administrative data. Elements of the study were deliberately pragmatic, seeking to reflect real-world clinical practice, such as surgeon determination of radiological severity. The study is limited in some respects by missing follow-up data. Automated reminders and an easy web-based data collection tool resulted in a 79% return rate from clinicians. However, PROM follow-up rates were generally low, reflecting the challenges faced in introducing multicentre research within a research-naïve clinical speciality. The completed PROMs demonstrated some evidence of selection bias, identified through a greater disease severity among those completing PROMs. We were also unable to investigate baseline predictors of outcome (i.e. disease severity) with PROMs. Nevertheless, this does not lessen the value of the PROMs in illustrating the impact of the disease on affected children.

The size of the SCFE population and variation in surgical pathways, coupled with an obvious difference in procedural safety and profound impact on the quality of life of the child, means that more definitive research is necessary. The research delivery network within UK paediatric orthopaedics has grown considerably since the initiation of this study, though this needs continued nurturing to ensure the feasibility of such research. In particular, a clinical trial must address the clear uncertainty related to severe stable SCFE to ascertain whether open reduction to correct deformity results in better patient outcomes than fixation without deformity correction. Furthermore, clinical trials and risk prediction models to guide the treatment of the opposite hip are needed, though given the paucity of events, this effort may need to be global to derive clinically meaningful results. In light of this study, we are pleased that the National Institute for Health Research have recently funded our group to conduct a RCT identifying the optimal treatment for severe stable SCFE.

In conclusion, this is a comprehensive cohort study of SCFE which has demonstrated that diagnostic delays were common and were associated with worse radiological deformity, also that education and innovative tools are a potential solution in order to maximize diagnostic opportunities. The experience of individual hospitals treating SCFE was limited, but mechanisms to consolidate care and learning throughout geographical regions may enhance care. We also found that there are several key aspects of treatment which may expose children to significant risk of complications and consideration should be given to them in the context of definitive RCTs to determine their efficacy.

**Take home message**

- Diagnostic delays in the treatment of slipped capital femoral epiphysis (SCFE) are common and associated with worse radiological deformity.

- The experience of individual hospitals is limited, and mechanisms to consolidate care and learning throughout geographical regions may enhance care.

- There are several uncertainties, relating to severe disease, unstable SCFE, and the unaffected hip, that warrant the consideration of definitive randomized controlled trials.

## References

[1] Perry DC , Metcalfe D , Costa ML , Van Staa T . A nationwide cohort study of slipped capital femoral epiphysis. Arch Dis Child. 2017;102(12):1132–1136. 10.1136/archdischild-2016-312328 28663349 PMC5754864

[2] Perry DC , Metcalfe D , Lane S , Turner S . Childhood obesity and slipped capital femoral epiphysis. Pediatrics. 2018;142(5):e20181067. 10.1542/peds.2018-1067 30348751

[3] Leunig M , Casillas MM , Hamlet M , et al. Slipped capital femoral epiphysis: early mechanical damage to the acetabular cartilage by a prominent femoral metaphysis. Acta Orthop Scand. 2000;71(4):370–375. 10.1080/000164700317393367 11028885

[4] Metcalfe D , Peterson N , Wilkinson JM , Perry DC . Temporal trends and survivorship of total hip arthroplasty in very young patients: a study using the National Joint Registry data set. Bone Joint J. 2018;100-B(10):1320–1329. 10.1302/0301-620X.100B10.BJJ-2017-1441.R2 30295530

[5] Kocher MS , Bishop JA , Weed B , et al. Delay in diagnosis of slipped capital femoral epiphysis. Pediatrics. 2004;113(4):e322-5. 10.1542/peds.113.4.e322 15060261

[6] Witbreuk M , Besselaar P , Eastwood D . Current practice in the management of acute/unstable slipped capital femoral epiphyses in the United Kingdom and the Netherlands: results of a survey of the membership of the British Society of Children’s Orthopaedic Surgery and the Werkgroep Kinder Orthopaedie. J Pediatr Orthop B. 2007;16(2):79–83. 10.1097/01.bpb.0000236234.64893.92 17273031

[7] Mooney JF 3rd , Sanders JO , Browne RH , et al. Management of unstable/acute slipped capital femoral epiphysis: results of a survey of the POSNA membership. J Pediatr Orthop. 2005;25(2):162–166. 10.1097/01.bpo.0000151058.47109.fe 15718894

[8] Sonnega RJA , van der Sluijs JA , Wainwright AM , Roposch A , Hefti F . Management of slipped capital femoral epiphysis: results of a survey of the members of the European Paediatric Orthopaedic Society. J Child Orthop. 2011;5(6):433–438. 10.1007/s11832-011-0375-x 22184504 PMC3221762

[9] Perry DC , Wright JG , Cooke S , et al. A consensus exercise identifying priorities for research into clinical effectiveness among children’s orthopaedic surgeons in the United Kingdom. Bone Joint J. 2018;100-B(5):680–684. 10.1302/0301-620X.100B5.BJJ-2018-0051 29701090 PMC6413768

[10] Vella-Baldacchino M , Perry DC , Roposch A , et al. Research priorities in children requiring elective surgery for conditions affecting the lower limbs: a James Lind Alliance Priority Setting Partnership. BMJ Open. 2019;9(12):e033233. 10.1136/bmjopen-2019-033233 PMC6955494 31892663

[11] Horton R . Surgical research or comic opera: questions, but few answers. Lancet. 1996;347(9007):984–985. 10.1016/s0140-6736(96)90137-3 8606606

[12] McCulloch P , Cook JA , Altman DG , Heneghan C , Diener MK , IDEAL Group . IDEAL framework for surgical innovation 1: the idea and development stages. BMJ. 2013;346:f3012. 10.1136/bmj.f3012 23778427 PMC3685515

[13] Ergina PL , Barkun JS , McCulloch P , Cook JA , Altman DG , IDEAL Group . IDEAL framework for surgical innovation 2: observational studies in the exploration and assessment stages. BMJ. 2013;346:f3011. 10.1136/bmj.f3011 23778426 PMC3685514

[14] Cook JA , McCulloch P , Blazeby JM , et al. IDEAL framework for surgical innovation 3: randomised controlled trials in the assessment stage and evaluations in the long term study stage. BMJ. 2013;346:f2820. 10.1136/bmj.f2820 23778425 PMC3685513

[15] Perry DC , Arch B , Appelbe D , Francis P , Spowart C , Knight M . A protocol for a nationwide multicentre, prospective surveillance cohort and nested-consented cohort to determine the incidence and clinical outcomes of slipped capital femoral epiphysis. Bone Jt Open. 2020;1(3):35–40. 10.1302/2633-1462.13.BJO-2020-0002 33215105 PMC7659633

[16] Perry DC , Arch B , Appelbe D , Francis P , Spowart C , Knight M . The BOSS Study. Determining the incidence and clinical outcomes of uncommon conditions and events in orthopaedic surgery. Bone Jt Open. 2020;1(3):41–46. 10.1302/2633-1462.13.BJO-2020-0008 33215106 PMC7659705

[17] Varni JW , Seid M , Kurtin PS . PedsQL 4.0: reliability and validity of the Pediatric Quality of Life Inventory version 4.0 generic core scales in healthy and patient populations. Med Care. 2001;39(8):800–812. 10.1097/00005650-200108000-00006 11468499

[18] Eidt-Koch D , Mittendorf T , Greiner W . Cross-sectional validity of the EQ-5D-Y as a generic health outcome instrument in children and adolescents with cystic fibrosis in Germany. BMC Pediatr. 2009;9:55. 10.1186/1471-2431-9-55 19715563 PMC2753333

[19] Wong DL , Baker CM . Pain in children: comparison of assessment scales. Pediatr Nurs. 1988;14(1):9–17. 3344163

[20] Bilbro NA , Hirst A , Paez A , et al. The IDEAL Reporting Guidelines: A Delphi Consensus Statement Stage Specific Recommendations for Reporting the Evaluation of Surgical Innovation. Ann Surg. 2021;273(1):82–85. 10.1097/SLA.0000000000004180 32649459

[21] No authors listed . Population estimates for the UK, England and Wales, Scotland and Northern Ireland: mid-2016. Office for National Statistics. https://www.ons.gov.uk/peoplepo pulationandcommunity/populationandmigration/populationestimates/bulletins/annu almidyearpopulationestimates/mid2016 (date last accessed 25 February 2022).

[22] Loder RT , Richards BS , Shapiro PS , Reznick LR , Aronson DD . Acute slipped capital femoral epiphysis: the importance of physeal stability. J Bone Joint Surg Am. 1993;75-A(8):1134–1140. 10.2106/00004623-199308000-00002 8354671

[23] No authors listed . International statistical classification of diseases and related health problems. 10th revision, 2nd edition. World Health Organization. 2004. https://apps.who.int/iris/bitstream/handle/10665/42980/9241546530_eng.pdf?sequence=1&isAllowed=y (date last accessed 1 March 2022).

[24] No authors listed . OPCS classification of interventions and procedures, version 4.8. Health and Social Care Information Centre, NHS. 2017. https://digital.nhs.uk/data-and-information/information-standards/information-standards-and-data-collections-including-extractions/publications-and-notifications/standards-and-collections/dcb0084-opcs-classification-of-interventions-and-procedures (date last accessed 1 March 2022).

[25] Rosenberg AR , Orellana L , Ullrich C , et al. Quality of life in children with advanced cancer: a report from the PediQUEST study. J Pain Symptom Manage. 2016;52(2):243–253. 10.1016/j.jpainsymman.2016.04.002 27220948 PMC4996729

[26] Tosounidis T , Stengel D , Kontakis G , Scott B , Templeton P , Giannoudis PV . Prognostic significance of stability in slipped upper femoral epiphysis: a systematic review and meta-analysis. J Pediatr. 2010;157(4):674–680. 10.1016/j.jpeds.2010.04.018 20605166

[27] Walton RDM , Martin E , Wright D , et al. The treatment of an unstable slipped capital femoral epiphysis by either intracapsular cuneiform osteotomy or pinning in situ: a comparative study. Bone Joint J. 2015;97-B(3):412–419. 10.1302/0301-620X.97B3.34430 25737527

[28] Kohno Y , Nakashima Y , Kitano T , et al. Is the timing of surgery associated with avascular necrosis after unstable slipped capital femoral epiphysis? A multicenter study. J Orthop Sci. 2017;22(1):112–115. 10.1016/j.jos.2016.08.012 27629912

[29] No authors listed . Open reduction of slipped capital femoral epiphysis. National Institute for Health and Care Excellence. 2015. https://www.nice.org.uk/guidance/ipg511/chapter/1-Recommendations (date last accessed 8 November 2019).

[30] Halverson SJ , Warhoover T , Mencio GA , Lovejoy SA , Martus JE , Schoenecker JG . Leptin elevation as a risk factor for slipped capital femoral epiphysis independent of obesity status. J Bone Joint Surg Am. 2017;99-A(10):865–872. 10.2106/JBJS.16.00718 PMC5426400 28509827

[31] Liu RW , Armstrong DG , Levine AD , Gilmore A , Thompson GH , Cooperman DR . An anatomic study of the epiphyseal tubercle and its importance in the pathogenesis of slipped capital femoral epiphysis. J Bone Joint Surg Am. 2013;95-A(6):e341-8. 10.2106/JBJS.L.00474 23515995

